# From stem to roots: Tissue engineering in endodontics

**DOI:** 10.4317/jced.50678

**Published:** 2012-02-01

**Authors:** Rita Chandki, M. Kala, Priyank Banthia, Ruchi Banthia

**Affiliations:** 1 M.D.S., Post graduate student. Department of conservative dentistry and Endodontics. Govt.Dental college and Research Institute, Bangalore, India; 2 M.D.S., Professor and Head. Department of conservative dentistry and Endodontics. Govt.Dental college and Research Institute, Bangalore, India; 3 M.D.S., Professor and Head. Department of Periodontics. Indraprastha dental college, Ghaziabad,India; 4 M.D.S., Professor. Department of Periodontics. Modern dental college and research center, Indore, India

## Abstract

The vitality of dentin-pulp complex is fundamental to the life of tooth and is a priority for targeting clinical management strategies. Loss of the tooth, jawbone or both, due to periodontal disease, dental caries, trauma or some genetic disorders, affects not only basic mouth functions but aesthetic appearance and quality of life. One novel approach to restore tooth structure is based on biology: regenerative endodontic procedure by application of tissue engineering. Regenerative endodontics is an exciting new concept that seeks to apply the advances in tissue engineering to the regeneration of the pulp-dentin complex. The basic logic behind this approach is that patient-specific tissue-derived cell populations can be used to functionally replace integral tooth tissues. The development of such ‘test tube teeth’ requires precise regulation of the regenerative events in order to achieve proper tooth size and shape, as well as the development of new technologies to facilitate these processes. This article provides an extensive review of literature on the concept of tissue engineering and its application in endodontics, providing an insight into the new developmental approaches on the horizon.

** Key words:**Regenerative, tissue engineering, stem cells, scaffold.

## Introduction

‘One can expect little progress from narrow, stereotyped thinking that fails to intelligently appraise and utilize the potential of improved treatment methods.’ - Dr Buonocore

With the clinical success rate of over 90%(1,2,3), millions of teeth are saved each year by root canal therapy. However, a significant number is still rendered unrestorable and doomed to extraction. It is a well established fact that people with dentofacial abnormalities experience social consequences including greater degrees of social avoidance and being perceived as possessing negative personality characteristics. (4)

Teeth are routinely replaced with conventional prostheses, i.e., removable prostheses, fixed dental prostheses, or implants. Denture therapy carries risk of complications such as denture-induced stomatitis, soft tissue hyperplasia, traumatic ulcers, altered taste perception and burning mouth syndrome(5). To overcome these disadvantages, the concept of osseointegration was introduced in the 1950s by Per-Ingvar Branemark, who observed the direct structural and functional bond formation between bone and titanium. However, osseointegration represents a direct connection between the implant and bone tissue and lacks the periodontium and cementum tissues present in naturally formed teeth, which function to cushion and modulate the mechanical stress of mastication (6). Hence, the need of an alterntaive restorative therapy, which may provide for a better substitute for natural teeth is inevitable. Undoubtedly, the best replacement for a natural tooth can only be a natural tooth.

The field of tissue engineering has literally exploded during the last decade, The knowledge generated through basic science research in the fields of stem cell biology, biomaterials (scaffolds), and morphogenetic signaling molecules coupled with recent advances in clinical research in the field of medicine, has resulted into an era where tissue engineering-based therapies are no more a farfetched dream but indeed a reality. Specifically talking about the dental field, years from now dental stem cells will hopefully be able to correct cleft palate sparing children from multiple surgeries, stem cells will also have the potential to save injured teeth and jaw bones, correct periodontal defects, and most strikingly regenerate entire tooth structure. This article reviews current status of the field of tissue engineering and its potential applications in dental sciences, specifically endodontics.

## Historical Perspective

The recognition that body parts may regenerate was first made in 330 BC by Aristotle, when he observed that a lizard could grow back the lost tip of its tail (7). Since then, study of regeneration has come a long way to find its applications in regenerative medicine and dentistry. We have moved from the surgical model of care to the medical model and are likely to move onto the biological model of care, seeking biological replacement for biological tissue.

Hermann (1952) was the first to carry out regenerative endodontic procedure, when he applied calcium hydroxide in vital pulp amputation (8). Later, Nygaard Ostby in 1961 evaluated a revascularization method for reestablishing a pulpdentin complex in permanent teeth with pulpal necrosis (9). His work was based on the known importance of the formation of a blood clot in wound healing and involved laceration of periapical tissue with an endodontic file. Subsequent regenerative dental procedures included guided tissue or guided bone regeneration (GTR, GBR) procedures and distraction osteogenesis (Block et al, 1995) (10) the application of platelet rich plasma (PRP) for bone augmentation (Kassolis et al, 2000) (11), emdogain for periodontal tissue regeneration (Heijl et al, 1997) (12), recombinant human bone morphogenic protein (rhBMP) for bone augmentation (Fjuimura et al, 1995) (13), and preclinical trials on the use of fibroblast growth factor 2 (FGF2) for periodontal tissue regeneration (Takayama et al, 2001; Lin et al, 2010) (14).

Nakahara (15) in 2006 proposed two potenetial methods for whole tooth regeneration.The first approach incorporates principles of tissue engineering, utilizing the inherent potential of stem cells to renew themselves and differentiate into different cell lineages when seeded onto appropriate scaffolds containing a combination of growth factors.The second approach involoves replicating the natural developmental processes of embryonic tooth formation.This could be achieved by transplanting artificial tooth germs into the bodies of appropriate animal hosts.

## Definitions

Regenerative Endodontics: Regenerative endodontic procedures can be defined as biologically based procedures designed to replace damaged structures, including dentin and root structures, as well as cells of the pulpdentin complex (16).

Tissue Engineering: An interdisciplinary field that applies the principles of engineering and life sciences toward the development of biological substitutes that restore, maintain, or improve tissue function (17).

Revascularization: Restoration of vascularity to a tissue or organ.

Apexogenesis: A vital pulp therapy procedure performed to encourage continued physiologic development and formation of the root end.

Apexification: A method to induce a calcified barrier in a root with an open apex or the continued apical development of an incompletely formed root in teeth with necrotic pulp tissue.

## Triad of Tissue Engineering

**Figure 1:**
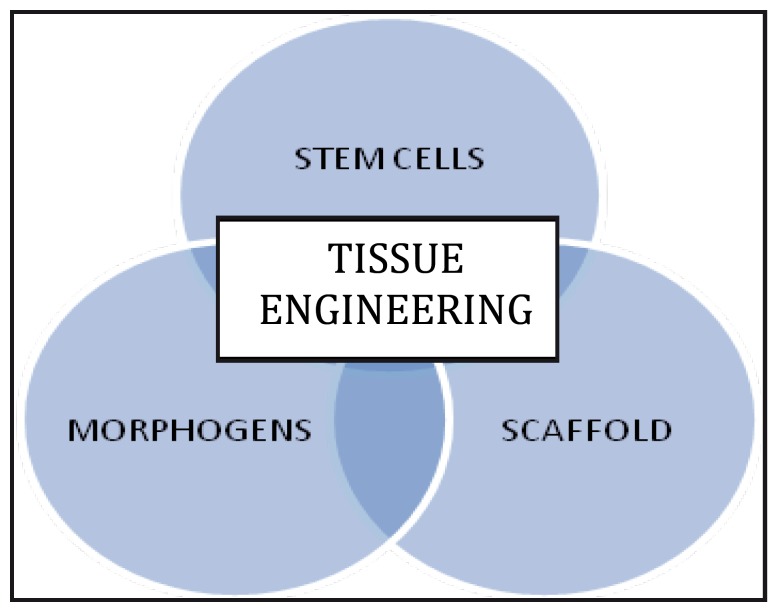
Triad of Tissue Engineering.

The interdisciplinary field of tissue engineering requires complex interactions between stem cells, morphogenetic signalling molecules and the scaffold or a matrix (Fig. 1).

Stem Cells

A stem cell is commonly defined as a cell that has the ability to continuously divide and produce progeny cells that differentiate (develop) into various other types of cells or tissues (18). Stem cells are the responsive cells of the triad of tissue engineering.

Classification of Stem Cells:

I. On the basis of origin.

a. Embryonic stem cells.

b. Somatic/ Adult/ Post-natal/ Mesenchymal stem cells. II. On the basis of Source.

a. Autologous: Obtained from same individual to whom they will be implanted.

b. Allogenic: Obtained from the donor of same species.

c. Xenogenic: Obtained from the donor of another species.

d. Syngenic/ Isogenic: Obtained from genetically identical organisms; twins, clones or highly inbred research animals.

III. On the basis of Potency (Range of differentiation).

a. Totipotent: can differentiate into all embryonic and extra embryonic cell types.

b. Pluripotent: can differentiate into all types of cells except cells of the embryonic membrane.

c. Multipotent: can differentiate into more than one mature cell.

d. Unipotent: Can differentiate into only one type of cells.

The specialized microenvironment, housing Adult stem cells and transient amplifying cells, forms a “niche”. In teeth, two different stem cell niches have been suggested: the cervical loop of rodent incisor for Epithelial Stem cells (19,20) and a perivascular niche in adult dental pulp for MSC (21). Following sources of stem cells have been recognized in human dental pulp:

1. Dental pulp stem cells (DPSCs; Gronthos et al, 2000)

2. Stem cells from Human exfoliated deciduous teeth (SHED; Miura et al, 2003)

3. Stem cells from apical papillae (SCAP; Sonoyama et al, 2006)

4. Periodontal ligament stem cells (PDLSCs; Seo et al, 2004)

5. Dental follicle progenitor cells.

Dental pulp stem cells:

The dental pulp contains a population of stem cells, called dental pulp stem cells (16), sometimes referred to as odontoblastoid cells, because these cells appear to synthesize and secrete dentin matrix like the odontoblast cells they replace (22). It is unknown where in the pulp the cells are recruited from. The cell-rich subodontoblast layer of Ho¨hl, perivascular cells or immature mesenchymal cells and fibroblasts have all been suggested (23). These cells were isolated for the first time from permanent third molars in 2000 by Gronthos et al.; these cells exhibited a differentiation potential for odontoblastic, adipogenic and neural cytotypes. In vitro DPSCs have been shown to produce sporadic but densely calcified nodules and when recombined with biodegradable scaffolds in vivo, they can form dentin–pulp-like tissues with an irregular shape (24,25). Further characterization has revealed that DPSCs are also capable of differentiating into other mesenchymal cell derivatives in vitro such as odontoblasts, adipoctyes, chondrocytes and osteoblasts.They have also been propsosed to possess putative immune-suppressive activity, which may be an advantage in cases of allogenic stem cell transplantation (26).

Stem cells from human exfoliated deciduous teeth (SHED).

Mesenchymal progenitors have been isolated from the pulp of human deciduous incisors. These cells were named SHED (Stem cells from Human Exfoliated Deciduous teeth) and exhibited a high plasticity since they could differentiate into neurons, adipocytes, osteoblasts and odontoblasts. In vivo SHED cells can induce bone or dentin formation but, in contrast to dental pulp, DPSC failed to produce a dentinpulp complex. SHEDs were different from DPSCs, affirming that they were ‘‘more immature’’: this probably because they were able to differentiate into a variety of cell types, to an extent greater than than DPSCs (27). SHED are distinct from DPSC with a greater proliferation rate and increased population doublings (28). Moreover, they offer painless stem cell collection with minimal invasion because they are retrieved from a tissue that is disposable and easily accessible.

Stem cells from apical papillae (SCAP)

Stem cells from the apical papilla are a population of multipotent stem cells isolated from the root apical papilla of human teeth. Compared with DPSC, SCAP have greater numbers of STRO-1 positive cells, faster proliferation a greater number of population doublings and increased capacity for in vivo dentine regeneration. Unlike DPSC and other MSC, SCAP are positive for telomerase activity which is present in embryonic stem cells and suggests a very immature source of cells available for hard tissue regeneration which has been demonstrated by the use of SCAP to engineer bioroots in minipigs (28). It has been suggested that SCAP might be the source of primary odontoblasts involved in the development of root dentine, in contrast to DPSC, which are most likely to be the source of replacement odontoblasts involved in reparative dentine formation (27).

Periodontal ligament stem cells (PDLSCs)

Stem cell population within the PDL was isolated and characterized by Seo et al. in 2004 (29). Periodontal ligament stem cells are more proliferative than BMSSC, with a longer lifespan, and higher number of population doublings in vitro. The potential of PDLSC to develop into other cell lineages and obtain periodontal ligament-like characteristics has been established by the ability of cultured PDLSC to differentiate into cementoblast-like cells, adipocytes and collagen-forming cells in vitro and the capacity to generate a cementum/PDL-like structure in vivo (29,30).

Dental follicle progenitor cells (DFPCS)

The dental follicle has long been considered a multipotent tissue, based on its ability to generate cementum, bone and PDL from the ectomesenchyme-derived fibrous tissue.These were first isolated by from the follicle of human impacted third molars (31). These cells can be maintained in culture for at least 15 passages. DFPCS can differentiate into cementoblasts in vitro (32) and are able to form cementum in vivo (33). Immortalized dental follicle cells are able to re-create a new periodontal ligament (PDL) after in vivo implantation (34).

Stem Cell Markers (24,27,29,31):

DPSCs: STRO-1, CD 146, STRO-4, Osteocalcin, Oct-4, Nanog, SSEA-3, SSEA-4,Nestin, TRA 1-60, TRA 1-81.

SHED: STR0-1, CD 146.

SCAP: DSP, BSP, ALP, CD 105.

PDLSCS: CD 146, CD 105, CD 166, STRO-1, MUC-18.

DFPCS: Notch-1, STRO-1, Nestin.

Techniques for Stem Cells Identification

(a) Staining the cells with specific antibody markers and using a flow cytometer, in a process called fluorescent antibody cell sorting (FACS).

(b) Immunomagnetic bead selection.

(c) Immunohistochemical staining.

(d) Physiological and histological criteria, including phenotype (appearance), chemotaxis, proliferation, differentiation, and mineralizing activity.

Morphogenetic Signalling Molecules/Growth Factors

Growth factors are extracellular secreted proteins that bind to cell receptors and modulate cellular activity eg by regulating the rate of proliferation, inducing differentiation into another cell type, or by stimulating cells to synthesize mineralizable matrices (35). Bone, cementum, PDL and apical papilla cells will proliferate and differentiate at different rates in response to growth factors and cytokines, but less clear, is a defined understanding of what pathways are involved in switching cells on. Primarily, four eminent families of growth factors appear to regualate the process of odontogenesis: Fibroblast growth factor, Hedgehog, Wingless( WNT) and Transforming growth factor. Dentin contains many proteins capable of stimulating tissue responses. Demineralization of the dental tissues can lead to the release of growth factors following the application of cavity etching agents, restorative materials, and even caries (36). Indeed, it is likely that much of the therapeutic effect of calcium hydroxide may be because of its extraction of growth factors from the dentin matrix (37). Once released, these growth factors may play key roles in signalling many of the events of tertiary dentinogenesis, a response of pulp-dentin repair (38). Extracts of dentin promote growth, because many growth factors are embedded into the dentin matrix during dentinogenesis. Interestingly, ethylenediaminetetraacetic acid (EDTA) very effectively releases growth factors from human dentin (39).

Scaffold:

Tissues are organized as three dimensional structures and appropriate scaffolding is necessary to provide a spatially correct position of cell location and to regulate differentiation, proliferation or metabolism. Extracellular matrix molecules control the differentiation of stem cells and an appropriate scaffold might selectively bind and localize cells, contain growth factors and undergo biodegradation over time. The seeding of cells on tissue engineering scaffolds is known as “creating a tissue construct”.

Requirements of a scaffold:

Effective for transport of nutrients, oxygen and waste.

Biocompatible and non-toxic.

Physically and mechanically strong.

Porous to allow cell placement, distribution and proliferation.

Permeability of culture medium.

In vivo vascularization ability.

Maintain osteoblastic cell phenotype.

Ease of fabrication.

Gradually degraded and replaced by regeneration tissue, retaining the features of the final tissue structure.

Scaffolds can be classified as natural or synthetic.The natural scaffolds are more biocompatible but Synthetic scaffolds offer improved control over physicochemical characteristics of the medium.They are more conducive to the growth of new tissue and relatively contraction free.Some of the commonly used scaffold materials have been summarized in the  Table 1.

**Table 1:**
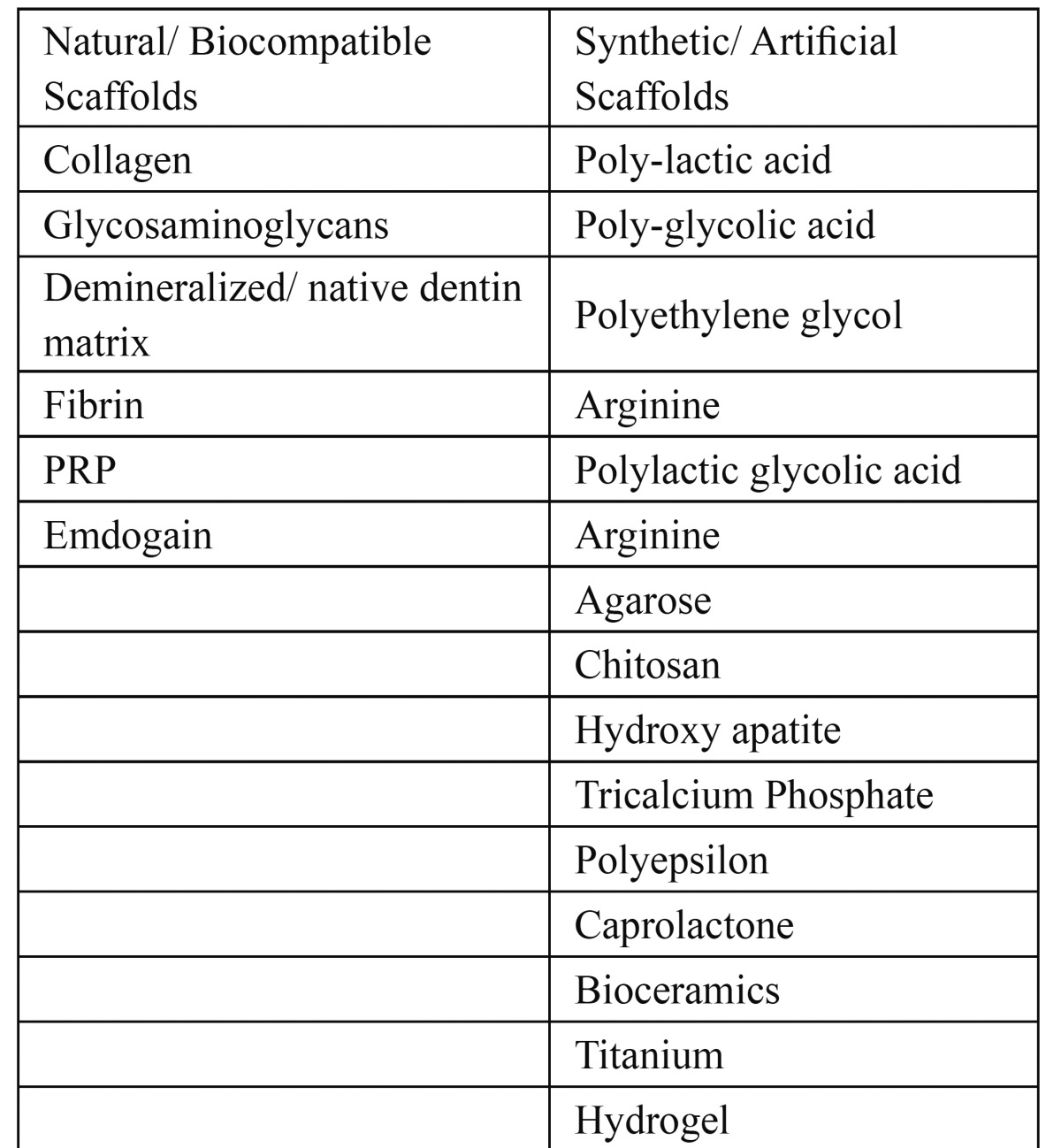
Examples of Natural and Artificial Scaffolds.

## Conclusion

The complete restoration of physiologic, structural & mechanical integrity of native pulp – dentin complex is ultimate goal of endodontic treatment.Till date, several approaches have been proposed to achieve this goal. For a new approach to gain acceptance, it should either produce better results or atleast equivalent results in lesser time and at a lower cost.Consequently, the road to tissue engineering is not smooth.The ethical and moral concerns regarding use of embryonic stem cells, challenges in identification of stem cells that can be maintained and expanded in culture and slower rate of human tooth embryogenesis are the barriers to be overcome for successful clinical application of concepts of tissue engineering in endodontics.Areas that might have application in development of regenerative endodontic techniques are(a) root canal revascularization via blood clotting (b) postnatal stem cell therapy (c) pulp implantation (d) scaffold implantation (e) injectable scaffold delivery (f) three dimensional cell printing (g) gene delivery. The future development of regenerative endodontic procedure will require a comprehensive research programme directed at each component and their clinical application. The regenerative therapy will revolutionize the future endodontics with synergistic confluence of advances in signaling pathways underlying morphogenesis & lineage of stem cells by morphogens such as BMPs, TGFβ & synthetic scaffolds.

## References

[1] Friedman S, Mor C (2004). The success of endodontic therapy: healing and functionality. J Calif Dent Assoc.

[2] Lazarski MP, Walker WA, Flores CM, Schindler WG, Hargreaves KM (2001). Epidemiological evaluation of the outcomes of nonsurgical root canal treatment in a large cohort of insured dental patients. J Endod.

[3] Salehrabi R, Rotstein I (2004). Endodontic treatment outcomes in a large patient population in the USA: an epidemiological study. J Endod.

[4] Newton JT, Fiske J, Foote O, Frances C, Loh IM, Radford DR (1999). Preliminary study of the impact of loss of part of the face and its prosthetic restoration. J Prosthet Dent.

[5] Holm-Pedersen P, Schultz-Larsen K, Christiansen N, Avlund K (2008). Tooth loss and subsequent disability and mortality in old age. J Am Geriatr Soc.

[6] Lin C, Dong QS, Wang L, Zhang JR, Wu LA, Liu BL (2009). Dental implants with the periodontium: a new approach for the restoration of missing teeth. Med Hypotheses.

[7] Nadig R Roopa (2009). Stem cell therapy- Hype or hope?. A review. J Conserv Dent.

[8] Herman BW (1952). On the reaction of the dental pulp to vital amputation and calxyl capping. Dtsch Zahnarztl Z.

[9] Nygaard-Ostby B (1971). Tiisue formation in the root canal following pulp removal. Scand J Dent Res.

[10] Block MS, Cervini D, Chang A, Gottsegen GB (1995). Anterior maxillary advancement using tooth-supported distraction osteogenesis. J Oral Maxillofac Surg.

[11] Kassolis JD, Rosen PS, Reynolds MA (2000). Alveolar ridge and sinus augmentation utilizing platelet-rich plasma in combination with freeze-dried bone allograft: case series. J Periodontol.

[12] Heijl L, Heden G, Svardstrom G, Ostgren A (1997). Enamel matrix derivative (EMDOGAIN) in the treatment of intrabony periodontal defects. J Clin Periodontol.

[13] Fujimura K, Bessho K, Kusumoto K, Ogawa Y, Iizuka T (1995). Experimental studies on bone inducing activity of composites of atelopeptide type I collagen as a carrier for ectopic osteoinduction by rhBMP-2. Biochem Biophys Res Commun.

[14] Takayama S, Murakami S, Shimabukuro Y, Kitamura M, Okada H (2001). Periodontal regeneration by FGF-2 (bFGF) in primate models. J Dent Res.

[15] Nakahara T (2006). A review of new developments in tissue engineering therapy for periodontitis. Dent Clin North Am.

[16] Murray PE, Garcia-Godoy F, Hargreaves KM (2007). Regenerative endodontics: a review of current status and a call for action. J Endod.

[17] Langer R, Vacanti JP (1993). Tissue engineering. Science.

[18] Rao MS (2004). Stem sense: a proposal for the classification of stem cells. Stem Cells Dev.

[19] Harada H, Kettunen P, Jung HS, Mustonen T, Wang YA, Thesleff I (1999). Localization of putative stem cells in dental epithelium and their association with Notch and FGF signaling. J Cell Biol.

[20] Mitsiadis TA, Barrandon O, Rochat A, Barrandon Y, De Bari C (2007). Stem cell niches in mammals. Exp Cell Res.

[21] Shi S, Gronthos S (2003). Perivascular niche of postnatal mesenchymal stem cells in human bone marrow and dental pulp. J Bone Miner Res.

[22] Kitasako Y, Shibata S, Pereira PN, Tagami J (2000). Short-term dentin bridging of mechanically- exposed pulps capped with adhesive resin systems. Oper Dent.

[23] Tziafas D, Smith AJ, Lesot H (2000). Designing new treatment strategies in vital pulp therapy. J Dent.

[24] Gronthos S, Mankani M, Brahim J, Robey PG, Shi S (2000). Postnatal human dental pulp stem cells (DPSCs) Proc. Natl Acad Sci USA.

[25] Gronthos S, Brahim J, Li W, Fisher LW, Cherman N, Boyde A (2002). Stem cell properties of human dental pulp stem cells. J Dent Res.

[26] Pierdomenico L, Bonsi L, Calvitti M, Rondelli D, Arpinati M, Chirumbolo G (2005). Multipotent mesenchymal stem cells with immunosuppressive activity can be easily isolated from dental pulp. Transplantation.

[27] Miura M, Gronthos S, Zhao M, Lu B, Fisher LW, Robey PG (2003). SHED: stem cells from human exfoliated deciduous teeth. Proc Natl Acad Sci USA.

[28] Friedlander LT, Cullinan MP, Love RM (2009). Dental stem cells and their potential role in apexogenesis and apexification. Int Endod J.

[29] Seo BM, Miura M, Gronthos S, Bartold PM, Batouli S, Brahim J (2004). Investigation of multipotent postnatal stem cells from human periodontal ligament. Lancet.

[30] Nagatomo K, Komaki M, Sekiya I, et al (2006). Stem cell properties of human periodontal ligament cells. Journal of Periodontal Research.

[31] Morsczeck C, Gotz W, Schierholz J, Zeilhofer F, Kuhn U, Mohl C (2005). Isolation of precursor cells (PCs) from human dental follicle of wisdom teeth. Matrix Biol.

[32] Kemoun P, Laurencin-Dalicieux S, Rue J, Farges JC, Gennero I, Conte-Auriol F (2007). Human dental follicle cells acquire cementoblast features under stimulation by BMP-2/-7 and enamel matrix derivatives (EMD) in vitro. Cell Tissue Res.

[33] Handa K, Saito M, Tsunoda A, Yamauchi M, Hattori S, Sato S (2002). Progenitor cells from dental follicle are able to form cementum matrix in vivo. Connect Tissue Res.

[34] Yokoi T, Saito M, Kiyono T, Iseki S, Kosaka K, Nishida E (2007). Establishment of immortalized dental follicle cells for generating periodontal ligament in vivo. Cell Tissue Res.

[35] Murphy WL, Simmons CA, Kaigler D, Mooney DJ (2004). Bone regeneration via a mineral substrate and induced angiogenesis. J Dent Res.

[36] Murray PE, Smith AJ (2002). Saving pulps: a biological basis. An overview. Prim Dent Care.

[37] Smith AJ, Cassidy N, Perry H, Begue-Kirn C, Ruch JV, Lesot H (1995). Reactionary dentinogenesis. Int J Dev Biol.

[38] Tziafas D (1995). Basic mechanisms of cytodifferentiation and dentinogenesis during dental pulp repair. Int J Dev Biol.

[39] Graham L, Cooper PR, Cassidy N, Nor JE, Sloan AJ, Smith AJ (2006). The effect of calcium hydroxide on solubilization of bioactive dentine matrix components. Biomaterials.

